# Social Cognition in Multiple Sclerosis: A Multidomain Investigation

**DOI:** 10.1155/bn/5973577

**Published:** 2026-02-03

**Authors:** Dalya Eda Akbalık, Murat Kürtüncü, Tuncay Gündüz, Mario Bonato

**Affiliations:** ^1^ Department of General Psychology and Padova Neuroscience Center, University of Padova, Padova, Italy, unipd.it; ^2^ Department of Neurology, Istanbul Faculty of Medicine, Istanbul University, Istanbul, Turkey, istanbul.edu.tr

**Keywords:** emotion recognition, multiple sclerosis, social cognition, theory of mind

## Abstract

While cognitive functioning in multiple sclerosis has been extensively studied, the presence of social cognition deficits in persons affected by relapsing–remitting multiple sclerosis (RRMS) remains comparatively less explored. This study recruited 30 Turkish patients with RRMS and 30 healthy controls (HCs) matched for demographic characteristics and cognitive status. We tested their social cognition abilities within two main subdomains, namely, theory of mind (ToM) and emotion recognition. The combination of tests we used was novel and rather broad as ToM was evaluated in both its affective and cognitive components while emotion recognition was evaluated in two tasks requiring either naming or discriminating facial expressions. All tests required verbal responses and were scored in terms of accuracy. The results indicated that patients with RRMS exhibited significantly lower accuracy compared to the HC group across all social cognition tasks. Correlation analyses revealed a significant negative relationship between emotion discrimination performance and both level of disability and disease duration. Additionally, RRMS patients showed significant deficits in recognizing negative emotions, while no differences with the HC group were observed for positive emotion recognition. The presence of a domain‐specific, selective deficit for social cognition was also confirmed after further controlling for overall cognitive functioning. Our findings capitalize on previous evidence demonstrating that social cognition impairments in RRMS have domain‐specific characteristics and can be reliably detected in a new cultural context.

## 1. Introduction

Multiple sclerosis (MS) is an inflammatory and degenerative neurological disorder affecting the central nervous system [1]. It is characterized by multifocal myelin sheath destruction and axonal loss, occurring over an unpredictable time course. Patients and their families suffer from significant physical and psychological burdens due to motor impairments and the presence of different degrees of cognitive dysfunctions [2]. Between 40% and 70% of MS patients have cognitive impairment, which has been reported in all stages and subtypes of the disease, including relapsing–remitting multiple sclerosis (RRMS) [3].

According to previous studies, patients with MS might also display deficits in social cognition (SC) [4–7]. SC is related to the mental processes that support social interactions, including the perception, interpretation, and response to other people’s intentions, tendencies, and behaviours [8]. Understanding emotional states is a domain of SC that requires distinguishing facial expressions and emotions [9]. Emerging evidence suggests that SC deficits in MS particularly concern emotional processing [10]. Beatty et al. conducted one of the first studies on MS patients’ emotion recognition abilities; patients performed poorly in cognitive tasks and were less accurate in distinguishing between facial emotions and identities than the healthy control (HC) group [11]. A similar study assessed the impact of MS on the ability to identify emotional and nonemotional information. The MS group showed impaired emotion recognition, but spared identity perception, suggesting a domain‐specific deficit [12]. Regarding emotion‐specific deficits, Prochnow et al. found impaired recognition for fear, surprise, anger, and sadness in MS patients, while no impairment was found for happiness and disgust [13].

In addition to emotion recognition, theory of mind (ToM) has been found to be pathological in MS. ToM represents another critical aspect of SC, and it refers to the ability to attribute the mental states of other people and use these attributions to understand and predict behavior of other persons [14]. ToM includes cognitive and affective domains; the cognitive aspect is the ability to reason and understand other individuals’ beliefs and intentions, while the affective aspect is the understanding and interpreting of these individuals’ emotions and feelings [15].

Under the assumption of domain specificity, also the presence of deficits in one or both components is highly debated as some authors only found deficits in cognitive but not affective ToM [16]. Roca et al. [17] came to a similar conclusion, yet they only used the FP test. On the other hand, Pöttgen et al. tested patients in their early stages of MS and observed an opposite pattern, with impairments mainly in the affective component of ToM as opposed to comparatively spared cognitive aspects [18]. Banati et al. [5] compared patients with early and late stages of MS and concluded for a different time course of the two types of SC deficits. While ToM affective components were impaired from the early stages, cognitive aspects of ToM tended to become pathological only in the later stages of MS [5].

The detection of SC deficits in RRMS has been widely described yet their nature is hotly debated. An overarching theme is whether they are core deficits or merely a consequence of the cognitive deficits. Recently, Wendt et al. [19] concluded that task‐based measures of mindreading (cognitive empathy) were highly correlated with general cognitive functioning. Also, they suggested that these measures might be capturing general cognitive ability or self‐perception rather than true social cognitive skills. More in general, the meta‐analysis by Baker et al. [20] found that performance on ToM tasks (especially RMET) is strongly associated with working memory, executive function, and processing speed. Also, studies on aging show that older adults often struggle with emotion recognition and ToM, but these declines parallel declines in executive function and processing speed rather than a domain‐specific impairment in SC [21].

Here, we present a preregistered study performed on a group of patients with RRMS. Parts of the current study are based on findings previously reported in the first author’s Master’s thesis [4].

Since the interaction between emotion recognition and ToM is crucial in enhancing interpersonal skills and this interaction significantly impacts social functioning [22], we assessed both these aspects of SC to precisely capture how social cognitive functioning is affected by MS. We also assessed in detail emotion recognition, including facial emotion identification (FEI) and discrimination. The combination of both tests is not often utilized in the existing literature. Additionally, we took into account several confounding factors like disability levels of RRMS patients through the Expanded Disability Status Scale (EDSS) score, disease duration, and general cognitive functioning. At the statistical level, we also controlled for MoCA scores to evaluate SC impairments independently of the influence of general cognitive functioning. This is the first study to investigate ToM abilities in a Turkish clinical MS cohort. Considering the cultural component of SC, its investigation in different countries/contexts is of paramount importance in order to understand its normal and pathological characteristics, including those in MS patients. We believe that these methodological characteristics may provide a rather comprehensive and reliable perspective on the relationship between SC in RRMS and other confounding factors listed previously.

According to previous evidence, we hypothesized that individuals with RRMS would exhibit significant deficits in facial emotion recognition and in ToM compared to HCs. Regarding the type of emotion, we anticipated more severe impairments in recognizing negative emotions compared to positive ones. Furthermore, we expected that longer disease duration and higher disorder severity would correlate with poorer social cognitive performance. Lastly, we hypothesized that better overall cognitive functioning, as measured by MoCA, would be positively associated with emotion recognition and ToM abilities.

## 2. Methods

### 2.1. Preregistration

The sample size, variables, hypotheses, and planned analyses for this study were preregistered on the Open Science Framework before data collection began (OSF link: https://osf.io/7umfe/?view_only=d4c151a10f164d80914a28bcb243a009). The link above also includes raw data and analysis scripts.

### 2.2. Participants and Procedure

This study was approved by the Clinical Research Ethics Committee of Istanbul Medical Faculty (Approval No. 2023/1557). All participants provided informed consent according to the declaration of Helsinki. The inclusion criteria (for both the experimental and control groups) were as follows: age between 18 and 65 years, having Turkish as a native language and (for the experimental group only) a low‐medium degree of disability (EDSS score between 0 and 6). Antidepressant use was allowed if the dosage was stable for 6 months, and patients with major psychiatric illnesses (e.g., substance abuse and schizophrenia), MoCA scores below 21 [23], recent MS attacks, or steroid treatment within the last 3 months were excluded. Additionally, patients scoring ≥ 17 on the Beck Depression Inventory or ≥ 16 on the Beck Anxiety Inventory were excluded to control for psychological effects on SC.

Sixty‐seven participants were initially tested for this study. Seven of them had to be excluded (see section 3.1). The final sample encompassed two groups: 30 RRMS patients and 30 HCs. RRMS patients were invited to participate during their clinical follow‐up at the Clinic for Central Nervous System Immunological Disorders at the Istanbul University Hospital. Healthy controls, matched for age, education, and gender, were recruited from hospital staff.

### 2.3. Neuropsychological Assessment

Participants were administered four SC tests to examine social cognitive functioning and the MoCA [24] test to assess global cognitive functioning. To examine affective and cognitive ToM abilities, two SC tests were used: Reading the Mind in the Eyes Test (RMET) [25] and the Faux Pas Recognition Test (FPRT) [26]. Other two SC tests were administered to assess participants’ facial emotion recognition abilities, including the FEI [27], and the Facial Emotion Discrimination (FED) Tests [27]. Furthermore, we administered Beck Depression Inventory (BDI) [28] and Beck Anxiety Inventory (BAI) [29]. These symptoms are widespread among MS patients; social‐cognitive deficiencies have been linked to the severity of depressive or anxiety symptoms [30]. A detailed description of each test is provided below.

#### 2.3.1. MoCA‐TR (Montreal Cognitive Assessment Scale in Turkish)

MoCA‐TR is a 10‐min screening test developed to briefly assess seven cognitive domains on a single page, specifically designed for detecting mild cognitive impairment. The test includes the following components: trail‐making test, cube copying, clock drawing, naming, counting forward and backward, vigilance, serial 7s, sentence repetition, verbal fluency, abstract thinking, delayed recall, and orientation [24].

The maximum score on the MoCA‐TR is 30. Scores above 21 are considered normal [23].

#### 2.3.2. FPRT

The FPRT is designed to assess individuals’ ability to interpret the thoughts and feelings of others in a socially inappropriate or awkward situation (faux pas). The adult version of the test was developed by Baron‐Cohen and colleagues in 1999 [26]. In this study, the short version consisting of 10 stories (five faux pas and five control stories) was used and each story was read to the participant, who was then asked six questions for each faux pas story. Five questions were asked to assess cognitive ToM abilities while one question was asked to assess the affective component of ToM [31]. In this study, the short version consisting of 10 stories was used. In 2021, Şandor and İşcen conducted a Turkish adaptation study in which they administered the FPRT test in both short and long versions to 420 healthy Turkish individuals. The short version of the test has three subsections; total scores for the correct detection of Faux Pas situations in FP stories (FPS) range between 0 and 5, total scores for answers to all questions in five FP stories (TFPS) range between 0 and 30, and total scores for the five control stories (TCS) range between 0 and 10 [31]. These subsections were prespecified measures based on the Turkish validation study of the FPRT [31].

In addition to the three subsections mentioned above, we wanted to perform exploratory statistical analysis for Question 4: Why do you think she/he said it? (question to interpreting intentions/called intentionality question in the current study), and Question 6: How do you think X felt? (question about empathy/named as affective ToM question in the present study). Scores for both questions range between 0 and 5.

#### 2.3.3. RMET

The RMET is a frequently used measure of affective ToM in both clinical and nonclinical populations [32]. The original version of the test includes 37 questions which have 36 actual questions and one test question [25]. Each picture presents one correct and three incorrect options to the participant. Throughout the test, participants are expected to choose the option that best describes what the person in each pair of eye pictures is thinking or feeling. A list containing 93 words, including expressions used in the test and words with similar meanings is provided to participants during the test. The list includes meanings and usage examples of the expressions [20]. In 2011, a Turkish validation and reliability study was conducted by Yıldırım and his colleagues on 117 healthy volunteers and they concluded that for clinical or non‐clinical samples, the 32‐item Turkish version of the eyes test can be useful in research on ToM and emotion regulation [33].

#### 2.3.4. FEI Test

Kerr and Neale developed two tests in 1993 to assess the perception of emotion expression in schizophrenia: the FEI test and the FED test [27]. The FEI test is presented as a slide show containing 19 black‐and‐white facial photographs depicting different emotional expressions. The photographs include six primary emotions (these are happiness, sadness, anger, fear, surprise, and shame). The test is arranged to display each photograph for 15 s, with a 10‐s interval between them. Participants are provided with a response key containing six basic emotions for each of the 19 items. As participants view each photograph, they are asked to mark which of the six basic emotions best describes what the person in the photo is feeling. A correct answer earns one point, and the maximum score for the test is 19. The Turkish validation and reliability study was done by Erol et al. in 2009 [34].

#### 2.3.5. FED Test

This test comprises 30 pairs of black‐and‐white photographs, each pair either displaying the same or different emotional expressions [27]. Similar to FEI, the test is presented as a slide show, with each pair shown for 15 s and a 10‐s interval between them. Participants are asked, for each photograph pair, whether the two faces show the same or different emotions. Participants indicate their choice (“same” or “different”) for each photograph pair, and 1 point is awarded for a correct answer (max score = 30). For this study, we used the Turkish validation and reliability study by Garcia et al. [35]. The rationale of introducing a discrimination task is that it should be even less taxing for higher cognitive functions than the identification test.

#### 2.3.6. BDI

The BDI [28] consists of 21 multiple‐choice questions to assess depressive symptoms, with scores ranging from 0 to 63. Symptoms are categorized as nonmild, mild–moderate, moderate–severe, or severe. For a study about its validity and reliability for the Turkish population, see [36].

#### 2.3.7. BAI

The BAI is a 21‐item screening tool, with each question scored from 0 (*none*) to 3 (*severe*). Scores of 8–15 indicate mild anxiety, 16–25 moderate anxiety, and 26–63 severe anxiety. Its validity and reliability for the Turkish population were established by Avcı [37].

#### 2.3.8. EDSS

The EDSS is the most widely used scale among patients with MS [38]. It encompasses all functional systems that may be affected by MS [39], and it is a highly effective method for measuring disability [40]. EDSS is characterized by a scoring system ranging from 0 to 10, in 20 steps (0.5 increments). “0” denotes a normal neurological examination, and “10” denotes an MS‐related death.

### 2.4. Statistical Analysis

The statistical analysis aimed to compare social cognitive functions between the RRMS and the HC group. Assumption checks were performed through Shapiro–Wilk test for four SC tests. For variables that violated normality assumptions we performed the Mann–Whitney *U* test (nonparametric), assessing whether the distributions of two independent groups differ. For the variables that did not violate normality assumptions, we performed an independent sample *t*‐test (parametric).

Multivariate analysis of variance (MANOVA) was performed on the outcome of the FEI test to examine differences in the accuracy of recognizing emotions, separately for positive and negative ones. Assumption checks for the MANOVA were assessed with the Shapiro–Wilk test and Box’s *M* test. Spearman’s correlation analysis assessed the relationships between RRMS severity (disease duration and EDSS score) and social cognitive functions. Each SC test (FPRT, RMET, FEI, and FED) was analyzed in its own independent multiple regression model with MoCA and group entered as predictors. Lastly, to rule out that the hypothesized difference was not due to unspecified cognitive deficits, we conducted a multivariate regression analysis entering MoCA scores as a covariate. Our goal was to evaluate how cognitive functioning influences performance on SC tests and to examine differences between the RRMS and HC groups while accounting for overall cognitive efficiency (MoCA).

JASP (JASP Team, 2024) was used for independent sample *t*‐test, Mann–Whitney *U* test, Pearson correlation, and ANOVA, while regression analyses were carried out in R (RStudio Team, 2020).

## 3. Results

### 3.1. Demographic and Clinical Features

Originally, 67 participants were tested for this study. Those who did not meet the cutoff criteria for MoCA, BDI and BAI were excluded, resulting in the removal of seven participants: four cases (two from the HC group and two from the RRMS group) due to MoCA scores below the inclusion cutoff, and three RRMS patients due to elevated depression and/or anxiety scores on the BDI or BAI. The two groups (RRMS and HC) were compared in terms of their anxiety and depression scores and no difference emerged (BDI: *U* = 466.0, *p* = 0.82; BAI: *U* = 513.0, *p* = 0.35). We also tested for correlations between SC measures and depressive/anxious symptomatology across participants, and no significant associations were found (*p* > 0.05).

The final clinical sample included 30 participants with RRMS. A group of 30 HCs was selected to precisely match the RRMS group in terms of age, years of education, gender (22 females and 8 males in each group), and MoCA scores (Table 1).

**Table 1:** Demographic and clinical characteristics of the study participants.

**Descriptive statistics**	**RRMS**	**HC**	**p**
Group size (*n*)	30	30	
Sex (F/M): *n* (%)	22/8 (73.3)	22/8 (73.3)	
Age (years) (mean ± SD)	45.3 (10.7)	44.8 (14.5)	0.85
Years of education (mean ± SD)	12.0 (4.7)	11.7 (3.9)	0.45
EDSS score (0–6) (mean ± SD)	2.5 (1.3)	—	—
Disease duration (years) (mean ± SD)	16.3 (8.1)	—	—
MoCA score (21–30) (mean ± SD)	26.0 (2.1)	26.0 (1.9)	1.0

Abbreviations: EDSS, Expanded Disability Status Scale; HC, healthy control; RRMS, relapsing–remitting multiple sclerosis.

### 3.2. Group Comparisons for SC Tests

Independent samples *t*‐tests were used for the RMET and FEI (Table 2) as normality assumptions were met, while for FPRT and FED (Table 3) normality was violated; therefore, Mann–Whitney *U* tests were used.

**Table 2:** Comparison of the RRMS and HC groups on RMET and FEI tests.

**SC tests**	**RRMS (** **M** **)**	**HC (** **M** **)**	**RRMS (SD)**	**HC (SD)**	**t**	**d** **f**	**p**	**Cohen’s** **d**
RMET	20.7	24.5	4.8	3.1	3.5	58	< 0.001	0.9
FEI	11.8	14.3	2.3	2.1	4.3	58	< 0.001	1.1

Abbreviations: df, degrees of freedom; FEI, facial emotion identification test; HC, healthy control; (M), mean; RMET, reading the mind in the eyes test, RRMS, relapsing–remitting multiple sclerosis; (SD), standard deviation; *t* value, independent samples *t* statistic.

**Table 3:** Comparison of the RRMS and HC groups on FPRT and FED tests.

**SC tests**	**RRMS (median)**	**HC (median)**	**U** **value**	**p**
FPRT	15.0	27.0	875.0	<0.001
FED	25.0	27.0	590.5	0.037

Abbreviations: FED, facial emotion discrimination test; FPRT, faux pas recognition test; HC, healthy control; RRMS, relapsing–remitting multiple sclerosis; *U* value, Mann–Whitney *U* statistic.

For RMET and FEI, Brown–Forsythe tests indicated homogeneity of variance (*p* > 0.05), so independent samples *t*‐tests (Student’s) were performed. Results indicate significant group differences on both tasks, with RRMS patients performing worse than HC participants (RMET: *p* < 0.001, FDR‐adjusted *p* = 0.004; FEI: *p* < 0.001, FDR‐adjusted *p* = 0.004).

The RRMS group performed significantly worse than the HC group on the FPRT (medians: 15.0 vs. 27.0, *p* < 0.001, FDR‐adjusted *p* = 0.004). Additionally, the RRMS group performed significantly worse than the HC group also on the fourth test, the FED (medians: 25.0 vs. 27.0, *p* = 0.037, FDR‐adjusted *p* = 0.037).

**Figure 1:**
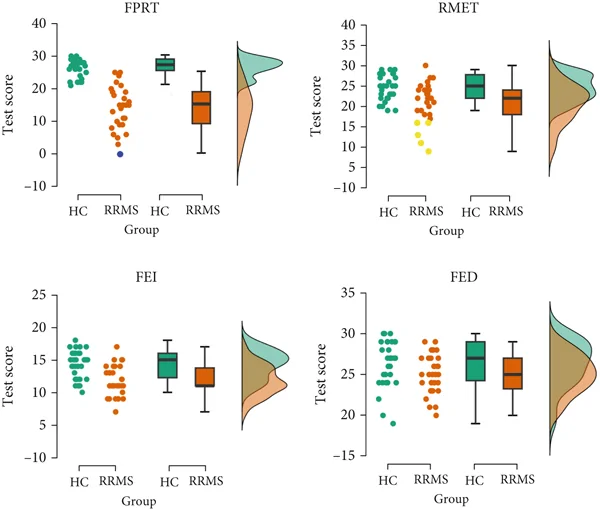
Performance for RRMS vs. controls across each SC test. HC, healthy control; RRMS, relapsing–remitting multiple sclerosis. Scores for HC and patients with RRMS are shown across the four social cognition tests (FPRT, RMET, FEI, and FED). Individual data points are displayed as scatter plots, while box plots represent each group’s median and interquartile range (first and third quartile), and raincloud plots illustrate the distribution of scores within each group. Two RRMS patients scored zero on the FPRT (blue point). One of them showed below‐average performance in both RMET and FED, whereas the other scored above average on these two tests. The yellow points in the RMET panel highlight the five patients who scored the lowest on this test. Four of them also had below‐average scores on the FPRT. Two of them scored below average in the FEI, and three in the FED test.

In short, significant group differences were found across all SC tests, with the RRMS group consistently demonstrating lower performance than the HC group (Figure 1).

### 3.3. FEI: Recognition of Positive and Negative Emotions

We developed the hypothesis of a dissociation between emotional valence based on prior findings in the literature suggesting that RRMS patients often present with specific difficulties in recognizing negative emotions compared to positive emotions in facial expressions, as assessed by the FEI test. Two MANOVA analyses were conducted; one for positive emotions with two dependent variables (surprise and happiness), and another for negative emotions with four dependent variables (fear, anger, sadness, and shame). For the fixed factors, we inserted groups (RRMS vs HC).

For negative emotions (sadness, fear, shame, and anger), the MANOVA revealed significantly lower scores for the RRMS group with respect to HCs, as shown by Pillai’s trace = 0.29, *F* (4, 55) = 5.79, *p* < 0.001 (shame: M = 0.43 vs. 0.53; fear: M = 0.49 vs. 0.66; sadness: M = 0.67 vs. 0.87; and anger: M = 0.61 vs. 0.77).

In contrast for positive emotions (happiness and surprise), no between groups significant difference emerged (Pillai’s trace = 0.02, *F* (2, 57) = 0.68, *p* = 0.511).

Emotion‐specific ANOVAs showed better performance for HC than for RRMs on test accuracy for three negative emotions: sadness, *F* (1, 58) = 10.706, *p* = 0.002; fear, *F* (1, 58) = 9.346, *p* = 0.003; and anger, *F* (1, 58) = 9.192, *p* = 0.004. On the other hand, performance did not differ between the groups for one negative emotion: shame, *F* (1, 58) = 1.631, *p* = 0.207.

Also, follow‐up univariate ANOVAs (Holm‐adjusted) revealed significant group differences for anger (pₕₒₗₘ  = 0.004), fear (pₕₒₗₘ  = 0.003), and for sadness (pₕₒₗₘ  = 0.002) but not for shame (pₕₒₗₘ  = 0.207).

Assumption checks revealed that all negative emotions in the MANOVA deviated from normality (Shapiro–Wilk test, all *p* < 0.05). Also, Box’s M was significant (*p* = 0.021), suggesting unequal covariance matrices. However, no extreme outliers were detected. According to Tabachnick and Fidell, Pillai’s trace can still be used as the test statistic within such a context, given its robustness under such conditions [41]. Also, Tabachnick and Fidell suggested that Box’s M is a highly sensitive test whose outcome is usually only taken into account when *p* < 0.001 and sample sizes are unequal [41]. Since the *p* value was greater than 0.01 with equal sample sizes, we still considered MANOVA as a robust test to assess negative emotion accuracy between the RRMS and HC groups. Complementary nonparametric analyses (Mann–Whitney *U* tests) yielded consistent results, supporting the robustness of the findings.

### 3.4. Correlations With Disease Duration and Disability

Spearman’s correlations were calculated to evaluate the relationship between the duration of the RRMS (disease duration in years) and performance on SC tests (FPRT, RMET, FEI, and FED). None of the SC tests revealed a significant correlation with disease duration; FPRT: rho = −0.144, *p* = 0.448, 95% CI [−0.48, 0.23], RMET: rho = −0.269, *p* = 0.150, 95% CI [−0.57, 0.10], FEI: rho = −0.174, *p* = 0.358, 95% CI [−0.50, 0.20], and FED: rho = −0.298, *p* = 0.110, 95% CI [−0.59, 0.07].

A second set of Spearman’s correlations investigated the relationship between the disability level of the RRMS patients (EDSS scores) and performance on SC tests (FPRT, RMET, FEI, and FED). Only the RMET test was significantly correlated with the EDSS score, showing higher disability levels associated with lower scores on the RMET test (negative correlation): rho = −0.425, *p* = 0.02, 95% CI [−0.68, −0.07]. Patients’ performance showed no significant correlation with EDSS scores for FPRT: rho = −0.119, *p* = 0.53, 95% CI [−0.46, 0.25], FED: rho = −0.285, *p* = 0.13, 95% CI [−0.58, 0.08], or FEI: rho = 0.087, *p* = 0.64, 95% CI [−0.28, 0.43].

### 3.5. Relationship Between SC and Global Cognitive Functioning

Multiple regressions were computed to investigate the relationship between each SC test and global cognitive functioning (Table 4). Crucially for our purposes, group differences in SC tests remained significant even after accounting for cognitive status. For the FPRT, the overall model was significant, *F* (2, 57) = 54.74, *p* < 0.001, explaining 65.8% of the variance (*R* ^2^ = 0.66, adjusted *R* ^2^ = 0.65). MoCA emerged as a significant positive predictor (*β* = 0.23, 95% CI [0.29, 1.55], *t* = 2.91, *p* = 0.005), whereas the RRMS group scored significantly lower than the control group (*β* = −0.78, 95% CI [−15.47, −10.33], *t* = −10.05, *p* < 0.001), even when MoCA performance was accounted for.

**Table 4:** Multiple and multivariate regression analyses for SC tests and MoCA scores.

**Dependent variable**	**Predictor**	**B**	**95% CI for** **B**	**SE** **B**	**β**	**p**
FPRT	MoCA	0.92	[0.29, 1.55]	0.31	0.23	0.005 ^∗∗^
	Groups	−12.90	[−15.47, −10.33]	1.28	−0.78	< 0.001 ^∗∗∗^
RMET	MoCA	1.12	[0.70, 1.55]	0.21	0.52	< 0.001 ^∗∗∗^
	Groups	−3.73	[−5.48, −2]	0.87	−0.42	< 0.001 ^∗∗∗^
FEI	MoCA	0.29	[0.02, 0.56]	0.14	0.23	0.038 ^∗^
	Groups	−2.50	[−3.6, −1.38]	0.56	−0.49	< 0.001 ^∗∗∗^
FED	MoCA	0.42	[0.09, 0.73]	0.16	0.32	0.011 ^∗^
	Groups	−1.30	[−2.6, −0.003]	0.65	−0.24	0.049 ^∗^
Multivariate results						
MoCA	Pillai’s trace = 0.38, *F* (4, 54) = 8.34, p < 0.001					
Groups	Pillai’s trace = 0.66, *F* (4, 54) = 25.89, *p* < 0.001					

*Note:* Multiple regression analyses include results of linear regression models for each SC test with MoCA and group as predictors. The multivariate results row presents the joint effect across all four dependent variables (FPRT, RMET, FEI, and FED), controlling for MoCA.

Abbreviations: *B*, unstandardized regression coefficient; SE *B*, standard error of the coefficient; **β**, standardized betas.

^∗^ *p* < 0.05,  ^∗∗^ *p* < 0.01, and  ^∗∗∗^ *p* < 0.001.

For the RMET, the model was significant, *F* (2, 57) = 23.08, *p* < 0.001, explaining 44.7% of the variance (*R* ^2^ = 0.45, adjusted *R* ^2^ = 0.43). Both MoCA (*β* = 0.52, 95% CI [0.70, 1.55], *t* = 5.27, *p* < 0.001) and group (*β* = −0.42, 95% CI [−5.48, −2], *t* = −4.29, *p* < 0.001) were significant predictors.

For the FEI, the overall model was significant, *F* (2, 57) = 12.24, *p* < 0.001, explaining 30% of the variance (*R* ^2^ = 0.30, adjusted *R* ^2^ = 0.28). MoCA (*β* = 0.23, 95% CI [0.02, 0.56], *t* = 2.13, *p* = 0.038) and Group (*β* = −0.49, 95% CI [−3.6, −1.38], *t* = −4.47, *p* < 0.001) both significantly predicted FEI performance.

Also, for FED, the model was significant, *F* (2, 57) = 5.43, *p* = 0.011, explaining 16% of the variance (*R* ^2^ = 0.16, adjusted *R* ^2^ = 0.13). MoCA (*β* = 0.32, 95% CI [0.09, 0.73], *t* = 2.61, *p* = 0.011) was a significant positive predictor, while the RRMS group again performed worse than the HC group (*β* = −0.24, 95% CI [−2.6, −0.003], *t* = −2.01, *p* = 0.049).

Lastly, we performed multivariate regression analyses using the MANCOVA function in R to ensure that the difference between RRMS and HC groups was still present while controlling for overall cognitive status (MoCA). Results revealed a significant multivariate effect between groups (Pillai’s trace = 0.7, *F* (4, 5) = 25.9, *p* < 0.001), indicating that RRMS patients and HCs differed in their overall SC profile even after adjusting for global cognitive performance.

### 3.6. Exploratory Analyses (FPRT and MoCA Subsections)

Independent samples *t*‐tests were conducted to compare RRMS and HC performance in FPRT subsections. As these analyses were not preregistered nor preplanned, they should be considered exploratory and interpreted with caution. For intentionality questions, the HC group (M = 4.60, SD = 0.68) performed significantly better than the RRMS group (M = 2.23, SD = 1.43), *t* (41) = 8.19, *p* < 0.001, *d* = 2.12. Similarly, for FP correct detection (FPS), the HC group (M = 4.83, SD = 0.38) demonstrated significantly better performance than the RRMS group (M = 3.17, SD = 1.46), *t* (32) = 6.04, *p* < 0.001, *d* = 1.56. For the affective ToM question, the HC group (M = 4.53, SD = 0.63) also scored significantly higher than the RRMS group (M = 2.83, SD = 1.53), *t* (38) = 5.62, *p* < 0.001, *d* = 1.45. Performance in the control stories (TCS) was at the ceiling for the HC group (M = 10.00, SD = 0.00) and nearly at the ceiling for the RRMS group (M = 9.73, SD = 1.02), allowing to further rule out that the deficit in SC was accompanied (or due to) the presence of rather unspecific cognitive impairments.

Additional analyses for MoCA subsections were performed to check whether there was a significant domain‐specific impairment in the RRMS group compared to the HC group. Also in this case, as these analyses were not pre‐registered nor a priori planned, they should be considered exploratory. Since the total MoCA scores of the two groups matched, we wanted to exclude the possibility that patients might compensate for poorer performance in specific domains (possibly, attention) with better performance in less affected ones (language, executive functions, visuospatial). To this aim, Mann–Whitney *U* tests were performed to test the difference between RRMS and HC groups’ performance on each MoCA subsection. The only significant difference was found in attention *U* (*N*_HC_ = 30, *N*_RRMS_ = 30) = 325.00, *p* < 0.035, with RRMS patients (median = 5.00) performing worse than HCs (median = 6.00). The other cognitive domains—executive function, memory, language, and visuospatial skill—did not show significant differences between the two groups.

We finally performed multivariate regression analyses to test whether the attention domain modulated performance on SC tests. The individual score in the MoCA attention section (digit span, vigilance and serial 7s) was entered as a covariate in four regression analyses for four SC tests. The group variable (RRMS vs. HC) was a significant predictor of FPRT (*t* = −9.05, *p* < 0.001), RMET (*t* = −3.25, *p* = 0.002), and FEI (*t* = 4.24, *p* < 0.001) scores, according to regression models; RRMS patients scored noticeably worse than controls. Nevertheless, none of the SC test results in the regression models were significantly predicted by performance in the MoCA attention section.

## 4. Discussion

Current literature on social cognitive functioning in adults with RRMS hotly debates whether their deficit is domain‐specific or rather a by‐product of subtle cognitive impairments. Moreover, previous studies often found impairments only in specific SC components. The main aim of this study was to empirically address these controversial aspects. We compared patients’ emotion recognition skills (particularly facial emotion recognition) and ToM (in both its affective and cognitive components) with those in a group of HCs matched for age, level of education, sex, and cognitive performance. Moreover, we tried to test whether and to what extent other factors such as disease duration, disability level, and MoCA scores modulate social cognitive functioning in RRMS groups. We did so by using tests which only measured accuracy and resorting to Turkish patients, a different population with respect to previous studies.

Our initial hypothesis was that persons with RRMS exhibited deficits in both facial emotion recognition abilities and ToM. Our hypothesis was fully confirmed by the impairments RRMS patients showed for both emotion recognition and ToM. To our knowledge, our approach was original as many previous studies only tested one of the two components while those focusing on both did not use the same tests we did.

For emotion recognition the RRMS group performed worse than the HC group in both FEI and FED. Already Berneiser et al. [30], reported that while MS patients were not impaired in facial identity discrimination, they consistently struggled with tasks requiring emotion recognition. Our study significantly extends this understanding by providing strong evidence of FED impairments in RRMS. We believe that assessing FED is essential, as it evaluates the ability to distinguish affective expressions without requiring explicit emotion labeling (naming), thereby reducing reliance on verbal abilities and likely on cognitive skills. Our findings indicate that deficits in RRMS extend beyond emotion labeling, suggesting disruptions in the fundamental processing of emotional cues, which may contribute to broader social communication difficulties.

Also, for ToM the deficits presented by RRMS patients were rather striking. On the RMET, which requires interpreting emotions from images of eyes, RRMS patients scored lower than HC. Similarly, studies by Garcia et al. [35], Charvet et al. [42], and Zahraie et al. [43] reported that MS patients performed worse than controls on the RMET, highlighting significant impairments in affective ToM. Furthermore, a substantial group difference was found in the FPRT total in the current study whereby RRMS patients had way fewer (about half) correct responses than the HC group. However, when it comes to the faux pas test subsections, Henry et al. discovered that patients performed considerably worse solely on intention‐related questions [44]. In contrast, Charvet et al. found a substantial difference between the MS and HC groups in terms of false belief and intention‐related questions [42].

In the current study, intentionality questions, affective ToM, and faux pas correct detection (FPS) revealed significant differences between the RRMS and HC groups. Our study extends the study by Henry et al. and by Charvet et al. thanks to the specific analysis for adding affective ToM questions; in this way, we not only assessed affective ToM through RMET but also evaluated it with the FPRT test.

However, the total score of control stories (TCS), which measures the ability to identify non‐Faux Pas stories (stories that do not include any social missteps), did not reveal any statistically significant difference between the two groups. This suggests that RRMS and HC participants were equally capable of recognizing situations where no social error occurred. The lack of significant differences in TCS performance indicates that the deficits in RRMS patients are rather specific to detecting and interpreting subtle social violations, rather than a general difficulty in processing the meaning of any story. This finding further supports the notion of a domain‐specificity for the impairment in SC we detected in RRMS.

According to previous literature, some studies found particular difficulty in recognizing negative emotions among MS patients compared to HCs. For instance, the study by Pitteri et al. described that MS patients might have selective difficulty in discriminating anger and fear [7]. In another study, Prochnow and colleagues found impairment in the recognition of facial expressions in all basic emotions except happiness and disgust for MS patients compared to the HC group [13]. In the current study, significant differences were observed in the recognition of fear, sadness, and anger, but not in shame, surprise, or happiness. Similarly, in Henry et al., recognition of fear and anger was disrupted for their MS group while no significant differences were found for recognition of surprise, happiness, sadness, or disgust [45].

Following the recommendations of Banati et al. [5], we tested the impact of disease duration, EDSS scores, and MoCA on the social cognitive functioning of RRMS patients. In addition, we controlled for MoCA to determine whether it significantly predicts deficits in SC in our RRMS cohort or if impairments in social cognitive functioning persist independently of its impact.

Firstly, we found no significant relationship between disease duration and most of the SC tests (FPRT, RMET, and FEI). A significant decline in FED scores was observed with increased disease duration. These findings align with Batista et al. [46] and Dulau et al. [47] who could not find a significant relationship between disease duration and any of the SC tests but contrast with Banati et al. [5], who found a link between RMET and longer disease duration with long‐term MS patients. We computed another correlation analysis to assess the relationship between the disability level of RRMS patients assessed through EDSS scores and performance on SC tests. Once again, a significant negative correlation was found only between the FED test and EDSS score.

Finally, we were interested in understanding to what extent overall cognitive functioning, measured through MoCA, would be able to account for pathological performance on SC tests. Our results confirmed significant positive correlations across all measures, showing that individuals with higher global cognitive function also tend to perform better on tasks requiring SC. This aligns with prior research on MS populations, such as studies by Montembeault et al. [48] and Sever Aktuna et al. [49], which identified a strong link between global cognitive abilities and facial emotion recognition. Also, the studies by Isernia et al. [50, 51] revealed that more efficient cognition is associated with better ToM abilities in MS patients. Still, the current study extends this understanding by demonstrating that even after accounting for general cognitive ability (MoCA scores), RRMS patients exhibit marked deficits in key SC domains compared to HCs. As deficits which were strikingly present in ToM abilities and emotion recognition tasks could be only in part accounted for by general cognitive abilities, we concluded for the presence of a domain‐specific impairment in SC.

Lastly, the observation that the average MoCA scores were the same between the RRMS and HC groups, yet the MoCA total score was significantly correlated with SC test performance, prompted us to further analyze the MoCA subsections. This analysis revealed that only the attention domain showed a significant difference between the two groups. Still, even this domain was not a significant predictor for the SC tests. These findings once again highlight the complexity of SC impairments in MS and suggest that such deficits may operate independently of general cognitive functioning. However, we need to consider the fact that the MoCA attention domain includes tasks such as digit span and serial 7s, which involve working memory and sustained attention. These abilities may support SC performance by helping individuals hold and process social information, and by managing the executive demands of ToM tasks. This overlap might suggest that attention and working memory could partly explain the link between cognitive and social deficits in RRMS. Future studies could explore these relationships more directly using targeted executive and memory measures.

One final issue we would like to mention refers to the possibility that some participants might have been better than others in counteracting their deficits. The concept of individual “compensation” has been used in the neurodevelopmental literature when accounting for individual differences in the field of SC deficits [52]. Future research should aim to investigate potential compensatory mechanisms, such as cognitive strategies or neural plasticity, that might enable some individuals to maintain social cognitive functioning despite underlying deficits. Understanding these aspects may provide useful information for intervention techniques targeted at reducing social cognitive impairments.

This study has several limitations that should be acknowledged. First, the relatively small sample size may limit the generalizability of the findings, and the cross‐sectional design prevents the establishment of causal relationships between disease progression and social cognitive decline. Second, while the study assessed key aspects of SC, it focused on specific tests like the Faux‐Pas Recognition Test and RMET, which may not capture the full scope of social cognitive abilities. Third, potential confounding variables such as fatigue and medication use, both common in RRMS, were not fully accounted for and may have influenced the results. Finally, although FPRT and RMET were adapted linguistically for the Turkish population, cultural differences in interpreting scenarios or facial expressions might still have introduced variability, potentially affecting the reliability of the findings.

In our study, participants who scored high in BDI and BAI were excluded to minimize the possible confounding factors of mood on social cognitive performance. While this approach provided a clearer and more reliable assessment of ToM and emotion recognition, we are aware that it can limit the generalizability of the findings to the broader RRMS population, in which emotional disturbances are common and often interact with cognitive functioning. Previous studies have shown that depressive and anxious symptoms can influence emotion recognition and ToM performance in MS including RRMS [2, 17, 18, 30]. However, the lack of significant correlations between mood symptoms and SC performance in our data further indicates that the observed group differences are unlikely to be influenced by these confounding factors.

## 5. Conclusion

In conclusion, the study shows that individuals with RRMS have significant impairments in SC, particularly in ToM and facial emotion recognition, compared to HCs. RRMS patients had more difficulty in recognizing negative emotions like fear, anger, and sadness. While overall cognitive functioning (MoCA scores) correlated positively with SC skills for the RRMS group, deficits in SC remained even after controlling for general cognitive ability, indicating a specific impairment in RRMS.

## Disclosure

The study here described has been performed as a Master’s thesis from D.E.A. (see References).

## Conflicts of Interest

The authors declare no conflicts of interest.

## Author Contributions

**Dalya Eda Akbalık:** conceptualization, data collection, visualization, writing – original draft, formal analysis, investigation. **Mario Bonato:** conceptualization, writing – review and editing, supervision, methodology, validation. **Murat Kürtüncü:** data collection, supervision, writing – review and editing, validation, methodology. **Tuncay Gündüz:** data collection, supervision, review and editing.

## Funding

No funding was received for this manuscript. Open access publishing was facilitated by Universita degli Studi di Padova, as part of the Wiley—CRUI‐CARE agreement.

## Data Availability

The raw data are available in a public repository (OSF): https://osf.io/7umfe/?view_only=d4c151a10f164d80914a28bcb243a009).
